# Effect of educational program on nurses’ performance regarding application of liberation bundle in pediatric intensive care unit

**DOI:** 10.1186/s12912-025-02821-7

**Published:** 2025-02-25

**Authors:** Reem Mohamed Seddik Ali, Asmaa Nasreldin Mosbeh, Mona Mohamed Hafez

**Affiliations:** 1 Pediatric Nursing Department, Faculty of Nursing, Damietta University, Dumyat, Egypt; 2 Pediatric Nursing Department, Faculty of Nursing, Ain Shams University, Cairo, Egypt

**Keywords:** Liberation bundle, Educational program, Nurses’ performance, Paediatric intensive care unit

## Abstract

**Background:**

Recent guidelines in paediatric critical care emphasize the implementation of the evidence-based liberation bundle to improve patient outcomes in paediatric intensive care units (PICUs). However, there is limited information on the application of this bundle in Egyptian hospitals, and the effectiveness of educational programs on nurse performance in this context remains unclear.

**Aim:**

This study aimed to evaluate the effect of an educational program on nurses’ performance regarding the application of the liberation bundle in paediatric intensive care unit.

**Methods:**

A one-group pre/post quasi-experimental design was employed. The study was conducted in the PICUs of Al-Azhar University Hospital and Menoufia University Hospital, involving a convenient sample of 52 paediatric nurses. Data were collected using two tools: a predesigned questionnaire to assess knowledge about the liberation bundle and an observational checklist to evaluate nurse practices before and after the educational program.

**Results:**

The results demonstrated significant improvements in nurses’ knowledge and practices post-intervention. The studied nurses’ total level of knowledge regarding the liberation bundle increased from 13.7 to 92.3% post-educational program X2 (P. value) = 89.143(0.000). The studied nurses’ total level of practices regarding the liberation bundle increased from 9.6 to 80.8% post-educational program X2 (P. value) = 89.143(0.000).

**Conclusion:**

The educational program significantly enhanced the nurses’ knowledge and practices in applying the liberation bundle in PICUs. This improvement in knowledge and practices is expected to lead to better outcomes for paediatric patients, including reduced mortality, shorter PICU stays, and fewer post-intensive care complications. By equipping nurses with the skills to implement the bundle, the program can improve recovery and long-term health outcomes in critically ill children.

## Introduction

The Paediatric Intensive Care Unit (PICU) provides critical care to infants, children, and adolescents with life-threatening illnesses or injuries [1]. Over the years, advancements in medical technology and paediatric critical care practices have significantly improved survival rates in PICUs worldwide [2]. However, surviving critical illnesses often comes with a host of post-discharge complications collectively referred to as Paediatric Post-Intensive Care Syndrome (PICS-p) [3]. PICS-p encompasses physical, psychological, cognitive, and social impairments that affect children’s long-term recovery and quality of life after they leave the PICU [4]. As paediatric survival rates rise, the focus has shifted toward minimizing these long-term complications and improving patient outcomes through evidence-based interventions [5].

In this context, the ABCDEF liberation bundle, developed by the Society of Critical Care Medicine (SCCM), has emerged as a comprehensive, evidence-based framework designed to enhance the care of critically ill patients in intensive care settings [6]. The liberation bundle aims to optimize multiple aspects of patient care by focusing on key elements such as pain management, sedation, delirium prevention, early mobility, and family engagement [7]. Numerous studies have shown that implementing the liberation bundle can significantly reduce mortality rates, PICU readmissions, and the occurrence of PICS-p symptoms [8–10]. However, despite the proven efficacy of this bundle in international settings, its implementation in Egypt, particularly in paediatric settings, remains limited.

In Egypt, the PICU system faces various challenges, including staffing shortages, limited access to ongoing training programs, and inconsistencies in the adoption of evidence-based practices [11]. These factors often result in suboptimal application of protocols such as the liberation bundle, which may be applied in fragments rather than as a holistic care approach. Additionally, cultural and resource limitations in Egyptian hospitals may further hinder the full-scale adoption of the liberation bundle [12]. This gap in practices underscores the importance of educational interventions aimed at enhancing the knowledge and practices of PICU nurses regarding the liberation bundle [13].

Nurses play a critical role in delivering patient-centred care in the PICU and are essential to the successful implementation of the liberation bundle [14]. Their knowledge, attitudes, and practices directly impact patient outcomes, especially in preventing PICS-p [15]. However, research on the knowledge and practices of PICU nurses regarding the liberation bundle in Egypt is sparse. Most existing studies have focused on adult patients or have examined different aspects of paediatric critical care without a comprehensive focus on the liberation bundle [16].

## Problem statement

Despite global advancements in paediatric critical care, Egyptian hospitals continue to face challenges in implementing evidence-based practice like the liberation bundle in their PICUs. The limited application of the bundle is particularly concerning, given its potential to reduce post-intensive care complications and improve overall patient outcomes [2]. In Egypt, where resources for continuous professional development and staff training are often scarce, many nurses lack adequate knowledge about the liberation bundle, resulting in fragmented and inconsistent care practices [17]. Moreover, the current body of research on the liberation bundle’s application in Egyptian PICUs is limited, making it difficult to identify the factors influencing its adoption and efficacy in local settings [18].

Studies conducted in Egypt have identified several barriers to the effective implementation of the liberation bundle. For instance, a lack of staff development programs, coupled with resource constraints, often prevents nurses from attending training courses that could improve their knowledge and application of the bundle [19]. Furthermore, many nurses report that they are unfamiliar with the full scope of the bundle’s components, such as spontaneous awakening and breathing trials, early mobility, and family engagement, all of which are crucial for minimizing the risk of PICS-p [20]. These gaps in knowledge and practices highlight the need for structured educational programs to improve the implementation of the liberation bundle in Egyptian PICUs.

Internationally, the liberation bundle has been widely adopted and proven effective in enhancing the quality of care in PICUs. Studies from the United States, Europe, and Asia have demonstrated that its implementation leads to significant reductions in PICU-related mortality, duration of mechanical ventilation, and the incidence of delirium and physical debilitation [21]. However, there is a noticeable gap in the literature when it comes to the application of the liberation bundle in low-resource settings like Egypt. This study aims to fill this gap by evaluating how an educational program can enhance the knowledge and practices of nurses in applying the liberation bundle in Egyptian PICUs.

## Local context and significance

Egypt’s healthcare system, particularly in paediatric critical care, faces distinct challenges, including high patient-to-nurse ratios, limited resources, and inadequate professional development opportunities for nurses [22]. These challenges are compounded by the lack of structured protocols for implementing comprehensive care frameworks like the liberation bundle. In the study settings, paediatric nurses often apply individual components of the bundle, such as pain management or sedation protocols, but fail to integrate these practices into a cohesive care strategy. This fragmented approach not only reduces the effectiveness of the bundle but also increases the likelihood of long-term complications in paediatric patients, including PICS-p [23, 24].

The significance of implementing the liberation bundle in Egyptian PICUs lies in its potential to improve patient outcomes by reducing the incidence of post-ICU complications. By providing nurses with the knowledge and skills necessary to apply the bundle in its entirety, this study seeks to promote more consistent, evidence-based care in Egyptian paediatric settings. Furthermore, the study’s findings could serve as a model for other low-resource countries facing similar challenges in critical care delivery. If successful, the educational program could be adopted by hospitals across Egypt, providing a framework for improving the overall quality of paediatric critical care in the region.

## Aim of the study

The current study aims to evaluate the effect of educational program on nurses’ performance regarding application of liberation bundle in paediatric intensive care unit.

### Research hypothesis

- The educational program would improve the nurses’ knowledge and practices regarding the application of the liberation bundle in the pediatric intensive care unit.

### Operational definition

#### Performance

in the current study performance include both knowledge and practices of nurses regarding caring of paediatric patients in emergency units (Ismaiel et al., 2022).

## Method

### Study design

This study employed a one-group pre/post quasi-experimental design to evaluate the impact of an educational program on nurses’ knowledge and practices in applying the liberation bundle in Paediatric Intensive Care Units (PICUs). A pre-intervention assessment was followed by the implementation of an educational program, with a post-intervention evaluation to determine changes in the nurses’ knowledge and practices.

### Study setting

The study was conducted in the PICUs of two hospitals in Egypt: Al-Azhar University Hospital in New Damietta and Menoufia University Hospital. Both hospitals provide specialized care for critically ill paediatric patients, with a focus on intensive care and the use of mechanical ventilation. Data collection and training sessions were held within the PICUs, where nurses provided direct patient care. The choice of these hospitals was based on their high patient load and the need for improved evidence-based practices in these units.

### Study subjects

The study involved a convenient sample of 52 paediatric nurses, 35 from the Menoufia University Hospital and 17 from Al-Azhar University Hospital. The sample size was calculated using G*Power (version 3.1.9.7), based on an effect size of 0.25, an alpha probability of 0.05, and a power of 0.85. Inclusion criteria were nurses with at least six months of experience in PICUs who provided direct care to paediatric patients. Nurses were selected for the study based on their availability during the study period, and no exclusion criteria were applied.

### Ethical considerations

Ethical approval for the study was obtained from the Scientific Research Ethics Committee of the Faculty of Nursing, Ain Shams University (Reference number: 45/2022). Written informed consent was obtained from all participants prior to data collection. Nurses were fully informed about the study’s objectives and were assured of the confidentiality of their responses and personal data. Participation was voluntary, and nurses had the right to withdraw from the study at any time without any consequences. Additionally, all ethical principles outlined in the Declaration of Helsinki were followed.

### Data collection tools

Data were collected using two primary tools: (1) a pre-designed questionnaire to assess the nurses’ knowledge regarding the liberation bundle and (2) an observational checklist to evaluate their practical application of the bundle.

### Knowledge questionnaire

The knowledge questionnaire was developed based on an extensive literature review of national and international studies on the liberation bundle [6, 25]. The questionnaire included 34 closed-ended questions that covered key areas such as pain assessment, sedation, delirium management, spontaneous awakening and breathing trials, early mobility, and family engagement. The questionnaire was initially developed in English and then translated into Arabic using a forward-backward translation method to ensure accuracy. Two independent translators conducted the forward translation, while a separate team, blind to the original version, performed the backward translation. This ensured that the final Arabic version maintained the original meaning and was culturally appropriate for the local context.

The questionnaire was administered to nurses in the PICUs during their shifts. To accommodate the rotating schedules, nurses were given the opportunity to complete the questionnaire during their breaks, ensuring minimal disruption to patient care. The average time to complete the questionnaire was 15 min. The scoring system was followed to assess the studied nurses’ knowledge regarding liberation bundle. The complete correct answer was scored “two marks”, the incomplete correct answer was scored “one mark” and the incorrect answer was scored “zero”. The total scores were summed up and converted into a percentage score. The total score of the knowledge regarding liberation bundle was 68 marks which equal 100% (100%) for total 34 questions. Then, the total score of the studied nurses’ knowledge was classified into two categories.

- “Satisfactory”: If the studied nurses’ total knowledge scores ≥ 85% which equals ≥ 57.8 marks.
- “Un satisfactory”: If the studied nurses’ total knowledge scores < 85% which equals < 57.8 marks.

### Observational checklist

An observational checklist was developed based on the standards set by the Society of Critical Care Medicine (SCCM) for the liberation bundle [26]. The checklist included six key components of the bundle: pain management, sedation, delirium assessment, spontaneous awakening and breathing trials, early mobility, and family engagement. The nurses’ practices were evaluated based on their adherence to these components before and after the educational program.

The observations were conducted by trained research assistants who were familiar with the PICU environment. Observers were not directly involved in the nurses’ routine work, minimizing bias. Nurses were aware that their practices were being observed, but they were not informed about the specific focus of the observations, reducing the risk of behaviour change due to awareness (Hawthorne effect). To further mitigate bias, observations were conducted over several shifts, ensuring that a variety of patient care scenarios were assessed. The scoring system was followed to assess the studied nurses’ actual level of practices regarding liberation bundle. The correctly done step was scored “two marks”, the incorrectly done step was scored “one mark” and the not done step was scored “zero”. The total scores were summed up and converted into a percentage score. The total score of the practices regarding liberation bundle was 40 marks which equal 100% (100%). Then, the total score of the studied nurses’ practices was classified into two categories.

- “Competent”: If the studied nurses’ total practices scores ≥ 90% which equals ≥ 36 marks.
- “Incompetent”: If the studied nurses’ total practices scores < 90% which equals < 36 marks.

### Instrument validity and reliability

A group of experts, including three professors and two assistant professors from the Pediatric Nursing Department at the Faculty of Nursing, Ain Shams University, modified and translated two instruments into Arabic and back into English. They examined the tools’ content validity, question types, and item clarity to ensure accuracy and prevent any errors in the study. The researchers used confirmatory factor analysis to validate the observational checklists for the liberation bundle and nurses’ knowledge regarding the liberation bundle. The Kaiser-Meyer-Olkin (KMO) and Bartlett Test of Sphericity were used to evaluate sample adequacy, with a Bartlett Test of Sphericity significance level of 0.05 and a minimum KMO value of 0.60 required. The results showed that the observational checklists and nurses’ knowledge scales had acceptable values for KMO and Bartlett’s Test of Sphericity, indicating their construct validity. The factor loadings for each construct exceeded the suggested level of 0.670, while the average variance extracted (AVE) values for all research variable dimensions were above 0.50, demonstrating convergent validity. The reliability of the tools was confirmed using Cronbach’s Alpha coefficient test, which showed the knowledge regarding the liberation bundle to have a reliability coefficient of 0.727 and the observational checklists for the liberation bundle to have a reliability coefficient of 0.873.

### Pilot study

Before the primary data collection, a preliminary study was conducted on a small group of 5 pediatric nurses, representing 10% of the total. The purpose was to test the study tools for their applicability, relevance, and the time needed to complete them.

### Educational intervention

The educational program was developed based on the initial assessment of nurses’ knowledge and practices and a review of recent literature. The program consisted of both theoretical and practical sessions and was designed to cover all aspects of the liberation bundle [8]. In the first session, the nurses defined the liberation bundle and explained its importance. In the second session, they discussed the items of the liberation bundle and the obstacles to its application in the PICU. The third session involved the application of various pain assessment scales and the discussion of measures to reduce pain in critically ill paediatric patients. The fourth and fifth sessions involved the performance of Spontaneous Awakening Trials (SATs) and Spontaneous Trials (SBTs) and the demonstration of the Richmond Agitation-Sedation Scale (RASS), respectively. The sixth session focused on early mobility and exercise, while the seventh session illustrated how to apply family engagement and empowerment. The final session defined the post-intensive care syndrome (PICS), its common causes, risk factors, and treatments, and illustrated common physical, psychological, and cognitive impairments.

Theoretical sessions lasted 15–20 min, while practical sessions lasted 30–45 min each. Although the time allocated for these sessions may appear brief, it was sufficient given that the nurses had some prior knowledge of the bundle, allowing the sessions to focus on filling knowledge gaps and emphasizing key concepts. Additionally, the material was supplemented by a detailed booklet in Arabic that nurses could review at their own pace.

The practical sessions involved hands-on demonstrations and role-playing to reinforce the skills needed to apply the liberation bundle in their clinical practices. To accommodate nurses with rotating shifts, the training sessions were scheduled during both morning and afternoon shifts, and nurses were divided into small groups (3–5 nurses per group) to ensure personalized attention and effective participation. The sessions were scheduled during less busy times in the PICU to avoid interference with patient care.

### Data collection procedures

Data collection occurred over six months, from May 2023 to October 2023. The researcher visited the two hospitals twice a week, on Saturdays at Al-Azhar University Hospital and Thursdays at Menoufia University Hospital. These days were selected based on the availability of nurses and to avoid overlap with busy periods in the hospitals, ensuring that patient care was not compromised. The timing of data collection was also coordinated with hospital management to align with staff scheduling and shift rotations.

The pre-intervention assessment involved administering the knowledge questionnaire and conducting observations of nurses’ practices before the educational intervention. After the completion of the educational program, a post-intervention assessment was conducted using the same tools to measure changes in knowledge and practices.

### Statistical analysis

Data were coded, organized, and analysed using the Statistical Package for the Social Sciences (SPSS) version 25. Descriptive statistics, including frequencies, means, and standard deviations, were used to summarize the demographic characteristics of the participants and their knowledge and practices scores. Pearson’s chi-square test and correlation coefficients were used to examine relationships between variables. Paired t-tests were used to compare pre- and post-intervention scores for knowledge and practices. A *p*-value of ≤ 0.05 was considered statistically significant.

## Results

Table 1 provides a comprehensive overview of the demographic characteristics of the 52 paediatric nurses who participated in the study. Nearly half (48.1%) of the nurses were aged between 20 and 25 years, while 30.8% were between 25 and 30 years, and 21.1% were 30 years or older. The mean age of the participants was 1.73 ± 0.79 years. In terms of gender distribution, the vast majority were female (84.6%), with males accounting for 15.4% of the sample.

Regarding educational background, most nurses (76.9%) had graduated from a technical institute of nursing, while 19.2% held a Bachelor of Nursing Science, and a small percentage (3.9%) had completed post-graduate studies. Experience levels varied, with 26.9% of nurses having between 1 and less than 3 years of experience, and the same percentage (26.9%) having 6 or more years of experience. Nurses with less than 1 year and between 3 and less than 6 years of experience each accounted for 23.1% of the sample. The mean years of experience were 2.54 ± 1.13 years.

**Table 1:** Distribution of the studied nurses according to their characteristics (*n* = 52)

Characteristics of the Studied Nurses	No.	%
**Age / Years**		
20: < 25	25	48.1
25: < 30	16	30.8
≥ 30	11	21.1
**Mean ± SD**	**1.73 ± 0.79**	
**Gender**		
Male	8	15.4
Female	44	84.6
**Level of Education**		
Technical Institute of Nursing	40	76.9
Bachelor of Nursing Science	10	19.2
Post-Graduate Studies	2	3.9
**Years of Experience**		
< 1 Year	12	23.1
1: < 3 Years	14	26.9
3: < 6 Years	12	23.1
≥ 6 Years	14	26.9
**Mean ± SD**	**2.54 ± 1.13**	
**Nurses According to their Pervious Attendance of Training Courses about Liberation Bundle**		
Yes	9	17.3
No	43	82.7

Additionally, the table reveals that a significant majority (82.7%) of the nurses had not previously attended any training courses related to the liberation bundle, while only 17.3% had attended such courses.

**Fig. 1:**
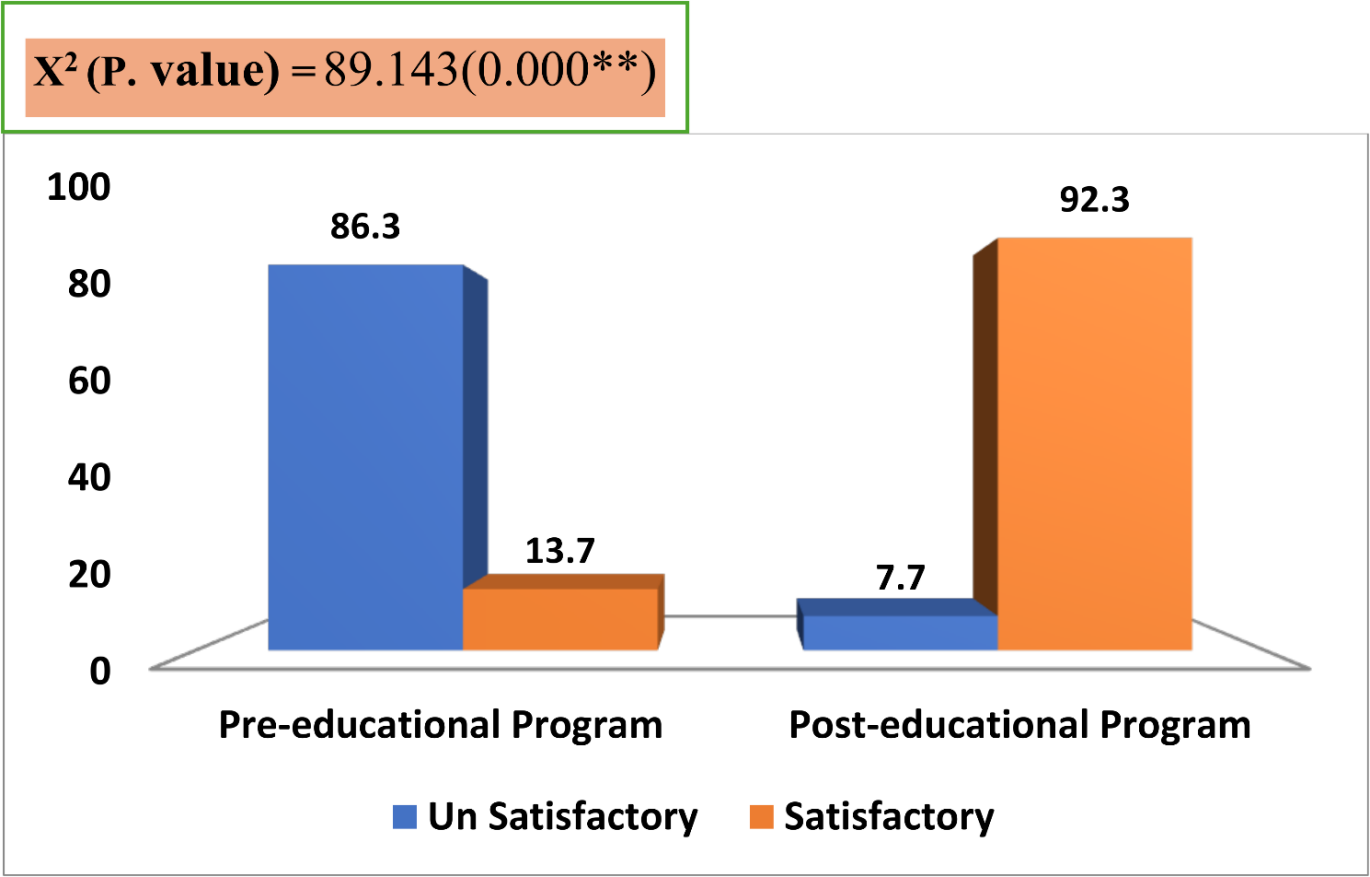
Percentage distribution of the studied nurses regarding their total level of knowledge about liberation bundle (*n* = 52)

Figure (1) clarified that the vast majority (92.3%) of the studied nurses had a satisfactory total level of knowledge regarding the liberation bundle post-educational program compared to only (13.7%) of them had satisfactory total level of knowledge pre-educational program. This reflected a statistically significant improvement in the studied nurses’ knowledge of the liberation bundle post-educational program.

**Fig. 2:**
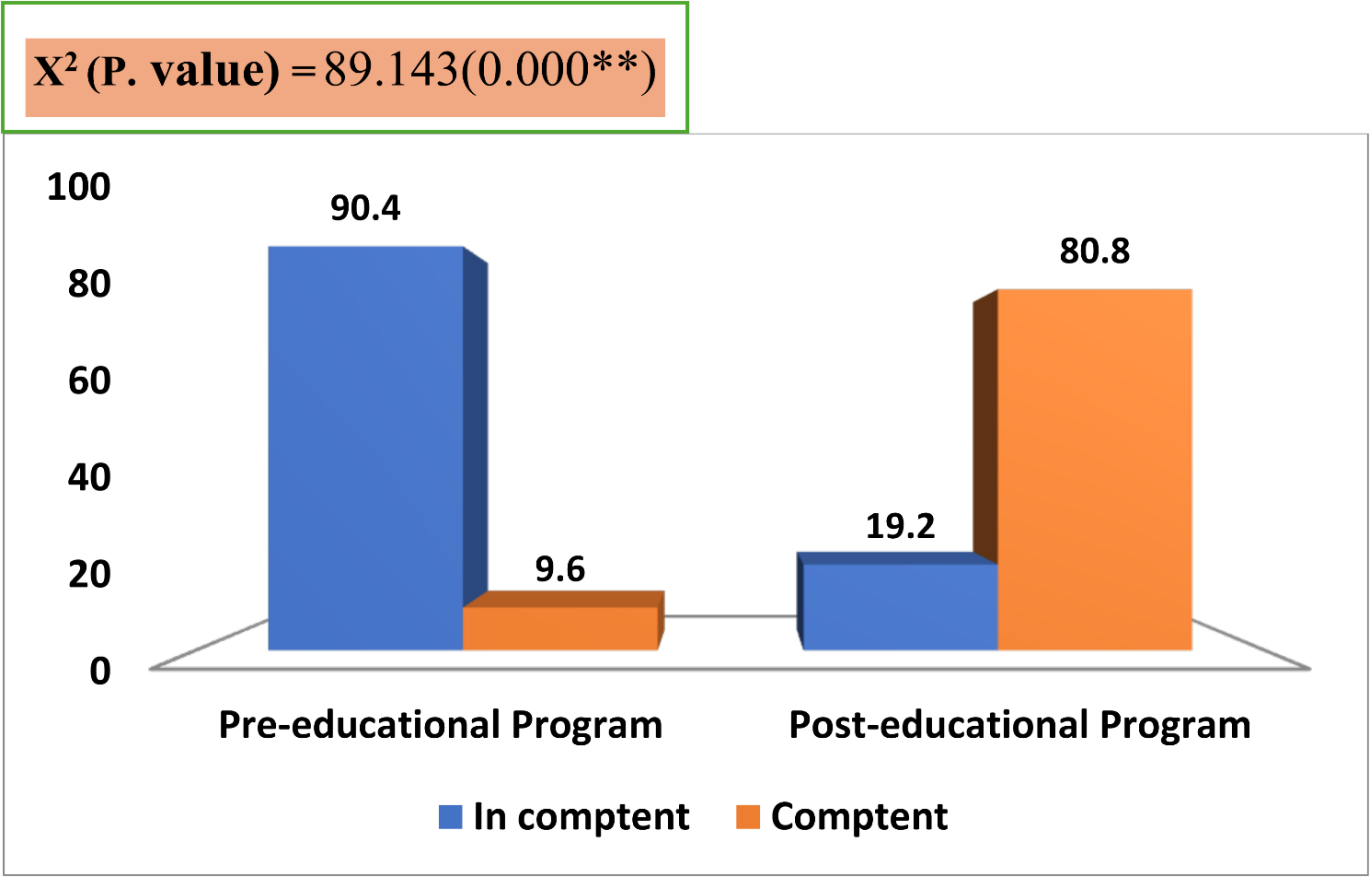
Percentage distribution of the studied nurses regarding their total level of practices about liberation bundle (*n* = 52)

Figure (2) clarified that the majority (80.8%) of the studied nurses had competent total level of practices regarding the liberation bundle post-educational program compared to only (9.6%) of them had competent total level of practices pre-educational program. This reflected a statistical improvement in the studied nurses’ total practices regarding the liberation bundle post-educational program.

**Table 2:** Relation between characteristics of the studied nurses & their total level of knowledge regarding liberation bundle post-educational program (*n* = 52)

Characteristics of the studied nurses	No.	Total knowledge post-educational program						X^2^ Sig.
		Un satisfactory			Satisfactory			
		No.		%	No.		%	
**Age / years**								
20: < 25	25 (48.1)	4		100.0	21		43.8	4.680 ^FE^ 0.129
25: < 30	16 (30.7)	0		0.0	16		33.3	
≥ 30	11 (21.2)	0		0.0	11		22.9	
**Gender**								
Male	8 (15.4)	0		0.0	8		16.7	^FE^ 1.000
Female	44 (84.6)	4		100.0	40		**83.3**	
**Level of education**								
Technical institute of nursing	40 (76.9)	4		100.0	36		**75.0**	1.053 ^FE^ 0.636
Bachelor of nursing science	10 (19.2)	0		0.0	10		20.8	
Post-graduate studies	2 (3.9)	0		0.0	2		4.2	
**Years of experience**								
< 1	12 (23.1)	3	75.0		9	18.8		5.077 ^FE^ 0.072
1: < 3	14 (26.9)	1	25.0		13	27.1		
3: < 6	12 (23.1)	0	0.0		12	25.0		
≥ 6	14 (26.9)	0	0.0		14	**29.2**		

* Statistically significant at *p* ≤ 0.05

Table (2) showed that there was no statistically significant relation between the characteristics of the studied nurses and their total level of knowledge regarding the liberation bundle post-educational program at the 5% level of statistical significance.

**Table 3:** Relation between characteristics of the studied nurses & their total level of practices post-educational program (*n* = 52)

Characteristics of the studied nurses	No.	Total practices post-educational program							X^2^ Sig.
		Incompetent				Competent			
		No.			%	No.		%	
**Age / years**									
20: < 25	25 (48.1)	6			60.0	19		45.2	3.328 ^FE^ 0.202
25: < 30	16 (30.7)	4			40.0	12		28.6	
≥ 30	11 (21.2)	0			0.0	11		26.2	
**Gender**									
Male	8 (15.4)	1			10.0	7		16.7	^FE^ 1.000
Female	44 (84.6)	9			90.0	35		**83.3**	
**Level of education**									
Technical institute of nursing	40 (76.9)	9			90.0	31		73.8	0.826 ^FE^ 0.785
Bachelor of nursing science	10 (19.2)	1			10.0	9		21.4	
Post-graduate studies	2 (3.9)	0			0.0	2		4.8	
**Years of experience**									
< 1	12 (23.1)		5	50.0			7	16.7	9.709 ^FE^ 0.011*
1: < 3	14 (26.9)		1	10.0			13	31.0	
3: < 6	12 (23.1)		4	40.0			8	19.0	
≥ 6	14 (26.9)		0	0.0			14	33.3	

* Statistically significant at *p* ≤ 0.05

Table (3) showed that there was no statistically significant relation between the characteristics of the studied nurses & their total level of practices regarding liberation bundle at a 5% level of statistical significance post-educational program related to age, gender as well as level of education (*p* = 0.202, 1.000 & 0.785) respectively. Also, there was a statistically significant relation between the characteristics of the studied nurses & their total level of practices regarding the liberation bundle post-educational program related to years of experience (*p* = 0.011).

**Table 4:** Correlation between the studied nurses’ total level of knowledge and total level of practices’ scores regarding liberation bundle (pre/ post-educational program) (*n* = 52)

Total level of practices	Total level of knowledge			
	Pre-educational program		Post-educational program	
	r	Sig.	r	Sig.
	0.609	0.000*	0.766	0.000*

* Statistically significant at *p* ≤ 0.05

Table (4) clarified that there was a positive correlation between the studied nurses’ total level of knowledge and total level of practice regarding the liberation bundle, with a statistically significant difference post-educational program (*r* = 0.766 & *p*-value = 0.000) compared to pre-educational program (*r* = 0.609 & *p*-value = 0.000). This means that their practice level improves when nurses acquire knowledge immediately after the educational program.

## Discussion

The findings of this study indicate a significant improvement in both the knowledge and practices of nurses regarding the liberation bundle following the educational intervention. This improvement underscores the importance of structured educational programs in enhancing evidence-based practice in paediatric intensive care settings. However, it is also essential to address conflicting findings from other studies and be deeper into the theoretical underpinnings that explain these improvements.

The improvement in both the knowledge and practices of nurses regarding the liberation bundle following the educational intervention might be due to the effective comprehensive educational sessions regarding the liberation bundle. These findings were in line with the studies conducted by Winnie et al. (2022) and Nardo et al. (2021), which reported that nurses’ total knowledge of all the items of the ABCDEF bundle improved after implementation an educational strategy about the bundle [27, 28].

While this study demonstrated clear improvements in both knowledge and practices, other studies have reported less favourable results. For example, a study by Ismaiel et al. (2022) found no significant improvement in nurses’ application of the ABCDEF bundle following educational interventions in PICUs [29]. These conflicting results could be due to differences in study design, particularly in terms of the intensity and duration of the educational programs [30]. In contrast to Ismaiel et al., our study provided more practical, hands-on sessions, which may have facilitated better integration of knowledge into practice.

Moreover, healthcare settings vary significantly in terms of resource availability and staff-to-patient ratios. Studies conducted in high-resource settings, where nurses have greater access to continuous professional development and better staffing, may not show the same degree of improvement post-intervention as studies conducted in resource-limited settings, such as Egypt [31]. In our study, the baseline level of liberation bundle application was low, leaving more room for noticeable improvement after training. Furthermore, disparities in nurse training programs across countries may account for some of these differences. Nurses in some regions may receive more comprehensive pre-service training on bundles like the ABCDEF bundle, resulting in less dramatic changes after post-service education [32].

The improvement in nurses’ knowledge and practices observed in this study can be explained through learning theories, particularly constructivist and adult learning theories [33, 34]. Constructivist theory posits that learners actively construct their knowledge based on their experiences and prior knowledge. In this context, the nurses’ pre-existing knowledge of critical care and their clinical experience served as the foundation upon which new concepts, like the liberation bundle, were integrated [35]. The educational program, which included group discussions, demonstrations, and practical sessions, allowed the nurses to contextualize this new information within their existing framework of knowledge, making it easier for them to apply the bundle in practice [36].

Adult learning theory (Knowles, 1984) further supports this improvement. Adult learners are typically self-directed and motivated by the relevance of the material to their professional lives [37]. In this study, nurses were able to immediately see the practical benefits of applying the liberation bundle, which likely enhanced their engagement and retention of the material. Additionally, adult learners prefer experiential learning, which was provided through practical sessions and role-playing in the educational program [38, 39]. This approach facilitated the nurses’ transition from merely understanding the theory behind the liberation bundle to actively applying it in their clinical practice.

The findings of this study have significant implications for the care of critically ill paediatric patients. Nurses who fully understand and apply the liberation bundle can markedly improve patient outcomes. The bundle addresses critical aspects of care, including pain management, sedation, delirium prevention, early mobility, and family engagement, all of which are essential for reducing the risk of Paediatric Post-Intensive Care Syndrome (PICS-p) [40]. By applying these principles, nurses can reduce the duration of mechanical ventilation, prevent delirium, and enhance early mobility, all of which contribute to faster recovery times and a reduction in long-term physical and cognitive impairments in paediatric patients [41, 42].

When nurses understand and use the bundle effectively, it leads to more consistent and comprehensive care. For instance, the spontaneous awakening and breathing trials promoted in the bundle help paediatric patients recover quicker from sedation and mechanical ventilation [43], which are associated with better long-term outcomes. Similarly, early mobility helps prevent the physical debilitation that is often a consequence of prolonged PICU stays. The application of family engagement strategies, another key component of the liberation bundle, ensures that parents or caregivers are involved in the decision-making process, which is crucial for the holistic recovery of paediatric patients [44].

The successful implementation of the liberation bundle is not solely dependent on nurses; it requires collaboration across the entire healthcare team [45]. This multidisciplinary approach is crucial for achieving the best outcomes for paediatric patients. Physicians, for example, play a vital role in leading spontaneous awakening trials and making decisions about sedation and analgesia [46]. Respiratory therapists are integral in coordinating spontaneous breathing trials, while physical therapists contribute to early mobility interventions. Pharmacists ensure appropriate medication management, particularly in choosing the right sedatives and analgesics that align with the goals of the liberation bundle [47].

In this study, the role of the multidisciplinary team was highlighted through the collaborative nature of the PICU. The educational program not only enhanced nurses’ knowledge and practices but also facilitated better communication and coordination among the healthcare team [45]. By integrating the liberation bundle into daily multidisciplinary rounds, the study promoted a more unified approach to patient care. This collaboration is essential, as the liberation bundle’s success hinges on its comprehensive application, which can only be achieved through teamwork [21].

The current study showed that, there was no statistically significant relation between characteristics of the studied nurses & their total knowledge regarding liberation bundle post-educational program. From the researcher point of view, this might be due to the studied nurses had more knowledge along with their characteristics.

The results of the present study differ from those of a study conducted by Bakhru et al. (2023), which discovered associations between the implementation of ABCDE protocols and the characteristics of the nurses [48]. Similarly, in contrast to the study findings, another study conducted by Huang et al. (2021) found significant differences in the scores of ABCDE bundle behaviour based on factors such as sex, occupation category, professional ranks and titles, working years in the hospital, working years in the PICU, regions of the hospital, and understanding of the ABCDE bundle [49].

The present study indicated no statistically significant relationship between the characteristics of the studied nurses and their overall practices, except for the years of experience. From the researcher’s perspective, this result may be attributed to the fact that more than three-quarters of the examined nurses graduated from a technical nursing institute and that the years of experience have enabled paediatric nurses to proficiently master skills. These findings are consistent with the results of a study conducted by Boehm et al. (2020), which found no association between the characteristics of the nurses and their practices [50].

The current study demonstrated a significant correlation between the overall level of knowledge and the level of practices related to the liberation bundle among the nurses under study post -educational intervention compared to pre -educational intervention.

From the researchers’ perspective, these pre-educational program results might be attributed to lack of continuous training and educational opportunities in the settings of the study, which could have hindered the improvement of the paediatric nurses’ level of practice. Additionally, equipment shortages, staff shortages, and a heavy workload might have contributed to the observed results pre the educational program. After the educational program, the studied nurses acquired knowledge regarding the liberation bundle and their level of practices also was improved.

These findings are consistent with the studies conducted by Fox & Tung (2022) and Mohamed et al. (2020), which found a positive correlation between the nurses’ overall knowledge and their practices related to the liberation bundle [51, 52].

### Limitations of the study

This study had several limitations that should be considered when interpreting the results. First, the lack of a control group and using a one-group quasi-experimental design weakens the ability to attribute the observed improvements in knowledge and practices solely to the educational intervention. Additionally, the convenience sampling method may limit the generalizability of the results, as the nurses who participated may not be representative of all PICU nurses. Future studies should employ a randomized controlled trial design and study two groups (study and control groups) to strengthen the validity of the findings.

Another important limitation is the absence of an assessment of the nurses’ attitudes towards the liberation bundle. Attitudes play a critical role in shaping behaviour and performance, and it is likely that the nurses’ motivation, confidence, and perceptions influenced how effectively they applied the bundle post-intervention. Future research should explore the impact of nurses’ attitudes on their performance to better understand the psychological factors that drive the application of evidence-based practice like the liberation bundle.

Lastly, while the study demonstrated a significant improvement in knowledge and practices, the long-term sustainability of these improvements was not assessed. Future studies should include follow-up evaluations to determine whether the benefits of the educational program persist over time.

### Recommendations

Based on the findings of this study, several recommendations can be made to enhance the implementation of the liberation bundle in Paediatric Intensive Care Units (PICUs). First, healthcare institutions should invest in continuous educational programs for nurses that focus on both theoretical and practical applications of the liberation bundle. These programs should incorporate adult learning principles, such as hands-on training and group discussions, to maximize engagement and knowledge retention. Additionally, future training should not only focus on knowledge and practices but also assess and address nurses’ attitudes toward evidence-based practice, as positive attitudes may further enhance the effectiveness of such programs.

A multidisciplinary approach is also recommended for the successful application of the liberation bundle. Collaborative efforts involving physicians, nurses, respiratory therapists, and other healthcare team members are crucial for achieving the best patient outcomes. Regular team-based training sessions and coordination between departments can improve communication and the comprehensive implementation of the bundle.

Finally, future research should focus on long-term follow-up studies to assess the sustainability of the improvements observed post-educational interventions. Including control groups in future studies will also provide stronger evidence for the effectiveness of such educational programs. Understanding the factors that influence the sustained use of the liberation bundle will contribute to the ongoing development of best practices in paediatric critical care.

## Conclusion

The findings of this study highlight the effectiveness of a structured educational program in improving nurses’ knowledge and practices regarding the liberation bundle in paediatric intensive care settings. By applying adult learning principles and practical hands-on training, the program significantly enhanced both theoretical understanding and practical application of the bundle, leading to better care for critically ill paediatric patients.

However, the role of nurses’ attitudes in their performance was not directly assessed, though it likely played an influential role. Future research should investigate the relationship between nurses’ attitudes, motivation, and the sustainability of performance improvements post-training. Understanding these factors may provide deeper insights into how to maintain high standards of care and improve patient outcomes over the long term.

Additionally, the absence of a control group in this study limits the ability to definitively link the educational program to the observed improvements. Future studies should include a control group and employ a randomized design to strengthen the findings. Overall, this study supports the need for ongoing education and a multidisciplinary approach to optimize the use of the liberation bundle in paediatric critical care, ultimately improving patient outcomes and reducing post-PICU complications.

## Data Availability

Data will be available from the authors on reasonable request.
